# Jefferson Medical College

**Published:** 1843-07-22

**Authors:** H. T. Child


### CLINICAL LECTURES AND REPORTS.

### JEFFERSON MEDICAL COLLEGE.

### CLINIC OF PROFESSOR MITCHELL.

*Dispensary of Jefferson Medical College, July* 12, 1843.

(Reported by H. T. Child.)

### LECTURE II.

Case I.—Julia McL——, æt 42. This case, gentlemen, is one of those difficult ones, which you will often meet with in practice; it is about the period of the "change of life" with her, and she has pains all over her system, she has head ache, she has a bad cough, &c. &c. You perceive she is of a melancholy temperament, (atra bilious of the ancients,) and her complaints are much more dependent upon mental than physical causes. You will find a disposition in such persons to magnify all the little ills that flesh is heir to—every pain is dwelt upon and thus increased—and as you know every one has a little pain, or headache, or some unpleasant sensation, which most persons pay little or no attention to, you see how easy it is for a person who is so disposed, to have a variety of complaints, for by dwelling upon these feelings others will arise, and the patient is harassed with a multitude of dreadful symptoms. Very often we find that those persons who become suddenly wealthy, in the fluctuations of mercantile business, are thus affected; for want of the accustomed exercise, they have little else to do but attend to their complaints, which are very generally increased by their diet and habits. This class affords the most profitable patients that a physician has; they will last him a life time very often. It is such persons as these that are so much benefitted by being sent to the fashionable watering places; and you will find that the advantage is in proportion to the amount of exercise they are compelled to take. The morning rambles and the evening entertainments at these places, prevent the indulgence of their idle habits, and the more difficulty they experience in getting to and from these places, the greater will be the advantage derived from such visits. The Sulphur Springs of Virginia afford these advantages; their distance, and the mountainous and hilly country which surround them make it a laborious task to visit them. An anecdote is related of Sydenham—he had a patient of this description under his care, and finding that his treatment was of little advantage, he determined to send his patient away from home. For this purpose he gave him a letter of introduction to a gentleman in Inverness, in the North of Scotland, who was to send him to a Dr. Robinson, of that place, and if the Doctor was not there he was to go immediately after him. He started off on horseback from London, and you may imagine the difficulties of such a journey in those days, when railroads and steamboats were not dreamed of. When he arrived at Inverness his friend informed him that Dr. Robinson was not there, but that he must go to Inverary, in the east of Scotland, to find him. Thus he travelled from place to place in search of Dr. Robinson, till at length he discovered that there was no such man in existence, that he was a creation of Dr. Sydenham's brain. By this time he had become very stout and well; he was determined to return immediately to London, and reek his vengeance on Sydenham. It is said that when he came into the presence of Sydenham, that although he was exceedingly angry, yet such was the benignity of the countenance of this great and good man—one of the greatest men that has ever adorned our profession—that he was restrained from violence, but he began to scold him. "Well," said the Doctor, "you went down to Inverness did you?" "Yes." "You went from there to Inverary, and you did not find Dr. Robinson?" "No! there is no such man, and you have deceived me." "Well, but how is your health?" "Oh! I am as well as ever I was." "Well," said the Doctor, "that is all I wanted. I have cured you, and you have learned this lesson, that if you would enjoy health you must take exercise with a motive."

It is necessary, in order to obtain the greatest advantage from exercise, that the mind should be interested. In this case the patient was determined to find Dr. Robinson, in hopes that he would cure him; when he discovered the deception he was so enraged that he lost no time in returning to London. We will give this woman small doses of ammonia sesqui carb. gr. ij. twice a day. If we could send her to the Virginia Springs, by the most difficult route, it would do her more good than any treatment here.

Case II.—Walter P——, æt. 33. Complains of pain in the left side, probably slight inflammation of the lung. His pulse is somewhat irregular. We will have him cupped over the seat of the pain, and give him pulvis ipecac. et. opii, gr. xvi. to night.

Case III.—Mary McK——, æt. 32. Has had pain in the head very often for six years. The attacks are frequently attended with a *sense* of fulness, vertigo, and other symptoms which might easily be mistaken for indications of plethora. This condition depends upon an undue developement of nervous excitability. The brain becomes so irritable that even less than the natural or healthy circulation causes a sense of oppression. This state of things is very apt to decieve a young practitioner, and he is disposed to resort to means to reduce the supposed fulness of blood. I was called, some years since, to see a butcher of this city, who had disease of the heart, attended with this kind of headache, for which he had been repeatedly bled, with an increase of the difficulty after each bleeding. Resort was had to a tonic plan of treatment, which soon relieved his head, though he died some time afterwards of organic disease of the heart.

*Causes.*—The predisposing cause is debility, which you know tends to increase the nervous excitability. The exciting cause in males, is most generally the influence of old carious teeth, and you will mostly find that when this sympathetic irritation exists, these teeth are seldom painful. Just as we see that when a person, with disease of the liver, has pain in the shoulder, he has none in the liver. I was called to see a young lady some time since who had been under treatment for periodical headache. She was of a highly nervous temperament, and had experienced no relief from the treatment of her physician. On examination I found several decayed teeth which were directed to be removed, and her headache was at once relieved. In females there is a powerful additional cause—the uterine system—in these cases you will generally find tenderness along some portion of the spine, when pressure is made there; these cases are more difficult to cure.

*Treatment.*—The removal of the cause, in the first instance alluded to, is generally sufficient. It may sometimes be necessary to make use of tonics, to overcome the state which has been induced by the long continued action of the exciting cause, and which will sometimes continue when the cause has ceased to exist. Where the disease depends upon a uterine affection, the treatment is not so simple, and must vary according to the nature of that affection. A tonic plan will generally be required.

In the case before us there are no indications of this affection. I find, on examination, that there are a number of carious teeth in her mouth; if these are removed she will probably be relieved at once.

Case IV.—William D——, æt. 32. Has had chronic pain in the stomach, at intervals for sixteen years; has slight tenderness on pressure; never vomits; he finds it worse in summer. This is a neuralgic affection. I think it probable some of the ganglia in the neighbourhood of the stomach are affected.

This patient might be benefitted by what Dr. Chapman facetiously called the "Punchinello Treatment." A gentleman in New York, named Halstead, had an affection very similar to that which our patient has, and as he was walking home one day from his store, he made a misstep in passing a gutter and experienced a severe shock in the stomach; this gave him considerable pain at the time, but he found that he ate his dinner with less inconvenience than he had for a long time been able to do. Pleased with the result he undertook the cure of cases of this kind. At first he was disposed to conceal his mode of treatment; but it was soon made public. His plan of punching and kneading the abdomen was extremely ludicrous, and has given rise to numerous jests. Many persons were benefitted by it, some entirely cured; but as Halstead had no knowledge of the different forms of disease, and was disposed to apply his mode of treatment to all, it soon proved fatal to some cases, and was abandoned. We may draw a useful lesson from this.

A gentleman of this city was, for some years unable to digest his food unless he rode on horseback immediately after eating. One day in riding, his horse stumbled and threw him, and he fell with the region of his stomach upon a knoll of grass,—he found that his disease was cured by the shock.

In the Sandwich Islands they treat neuralgia by pressing, kneading, and pounding the parts. While I was in Canton some years ago, a vessel from these Islands was there, and being affected with nervous rheumatism of the arm and shoulder, the Sandwich Surgeon proposed to *champ foo* me; he commenced by gently pressing and tapping the affected part, this was gradually increased till his blows were severe; in less than an hour the pain was gone.

I find the effect of an emetic often answer a good purpose, perhaps mechanically and physiologically, and will prescribe for this patient an emetic of cupri sulphas, gr. v., pulv. ipecac. gr. xx.

Case V.—Boyd W——, æt. 26. Complains of pain in the breast, has a cough, and discharged (he thinks by emesis) a considerable amount of blood; says he is not of a consumptive family. On examination there appears to be some indication of an affection of the upper lobe of the left lung. He was directed to take nitric acid and an infusion of wild cherry bark, and to exercise in the country.

Case VI.—Mary E——, the 11th case of last week. Has discharged a large quantity of worms, which she says were about the thickness of a darning needle, and were knotted together in a ball, the size of a hen's egg; complains at present of a cough, for which she was directed to take brown mixture.

Note.—There were several cases of influenza at the clinic to-day. The weather being bad, fewer cases were present than usual.

